# Comprehensive next‐generation profiling of clonal hematopoiesis in cancer patients using paired tumor‐blood sequencing for guiding personalized therapies

**DOI:** 10.1002/ctm2.222

**Published:** 2020-11-10

**Authors:** Ziyang Li, Wensou Huang, Jiani C. Yin, Chenglong Na, Xue Wu, Yang Shao, Huaxin Ding, Jinming Li

**Affiliations:** ^1^ National Center for Clinical Laboratories Beijing Hospital National Center of Gerontology Beijing China; ^2^ Department of Minimally Invasive Interventional Radiology The 2nd Affiliated Hospital of Guangzhou Medical University Guangzhou China; ^3^ Nanjing Geneseeq Technology Inc. Nanjing China; ^4^ School of Public Health Nanjing Medical University Nanjing China; ^5^ The Ningbo Diagnostic Pathology Center Ningbo China

Dear Editor:

In this study, we report for the first time the landscape and clinical relevance of clonal hematopoiesis (CH) in Chinese cancer patients using next‐generation sequencing (NGS) and emphasize the need for paired tumor‐blood sequencing to better inform clinical actions given the high prevalence and largely nonrecurrent nature of CH mutations.

CH is a common aging‐associated process that involves the accumulation of leukemia‐associated somatic mutations in hematopoietic stem cells, which can lead to clonal expansion and predispose to hematologic malignancies.^1^, ^2^, ^3^, ^4^, ^5^ The broad use of NGS technology in detecting oncogenic mutations has led to the growing recognition of CH‐associated mutations that are often present in the blood and, as a consequence of infiltrating leukocytes, the tumor tissue from patients with solid tumors.^5^, ^6^, ^7^, ^8^ Therefore, it is necessary to evaluate the incidence and clinical implications of CH as it could confound the results of tumor profiling. However, the significance of CH in the Chinese population has not been examined.

Here, we performed NGS on matched tumor‐blood samples from 4,544 patients (median age at DNA sampling, 60 years [range: 4‐91]; 55.5% males) with diverse solid tumors (Table S1 and Figure 1A) using a targeted 425‐gene panel (GeneseeqPrime®), which includes 44 hematologic malignancy‐associated genes (Table S2) in a CLIA‐ and CAP‐accredited laboratory (Nanjing Geneseeq Technology Inc., China) from May 2017 to April 2019. CH was defined as nonsynonymous mutations in which the blood variant allele fraction (VAF) is ≥2× that in the respective tumor.^5^

**FIGURE 1:**
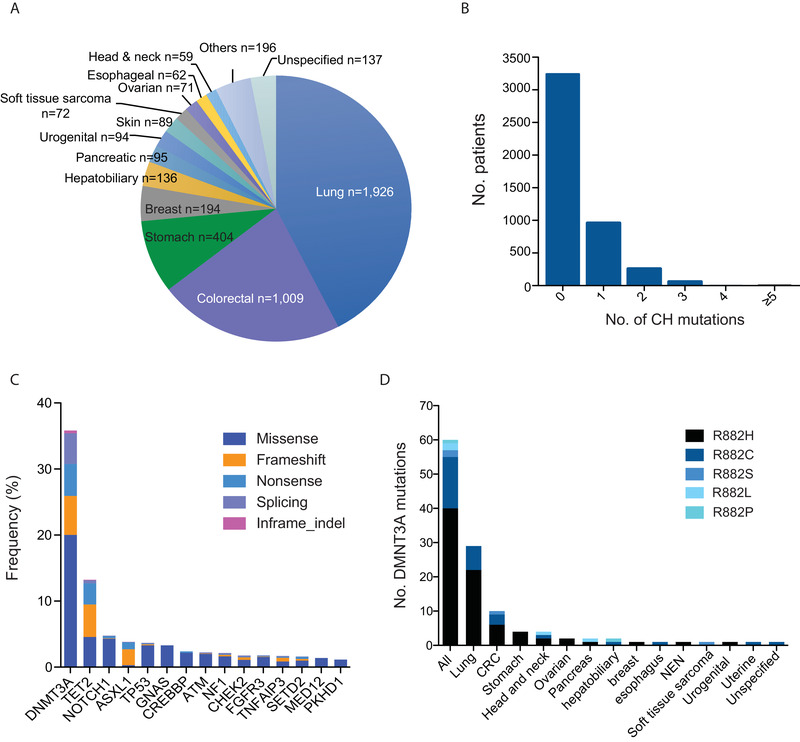
**Distribution of CH variants across patients with diverse solid tumor types. A**, Distribution of cancer types in the study. **B**, No. of CH alterations in each patient. **C**, Distribution of different types of mutations among the top 15 recurrent CH genes. **D**, *DNMT3A* R882 “hot‐spot” mutations across various cancer types

A total of 1,749 CH variants in 237 genes were identified in 1,301 (28.6%) patients, of which 26% carried more than one CH mutations (Figure 1B and Table S3). While the majority of CH mutations were nonrecurrent, a high frequency of *DNMT3A* (35.8%), *TET2* (13.2%), *ASXL1* (3.8%), and *TP53* (3.7%) alterations were identified (Figure 1C). By contrast, two other commonly reported CH‐associated genes, *JAK2* (0.8%) and *SF3B1* (0.8%), were found at low frequencies. Alterations in *DNMT3A*, *TET2*, and *ASXL1* were largely disruptive (Figure 1C and Figure S1A‐C). The R882 hotspot accounted for 11.5% of *DNMT3A* mutations. Previous reports of CH show almost exclusive R882H and few incidence of R882C in *DNMT3A*.^4^, ^5^ In our cohort, we found a diverse array of R882 variants (Figure 1D). Notably, we also found a high frequency of *NOTCH1* alterations (Figure 1C and Figure S1D), with ∼60% reported in various cancers (COSMIC database), 26% of which were associated with hematologic malignancies.

Consistent with previous studies,^4^, ^5^, ^6^ we observed an increased incidence of CH with age overall (Figure 2A) and across cancers (Figure S2). Different cancer types varied in CH prevalence, which is likely attributable to differences in age of disease onset (Figure S2). Interestingly, aging was also associated with accumulation of CH (Figure 2B) and increases in mutational frequencies of individual CH genes (Figure 2C). Consistent with their role as early genes in hematologic disease,^9^ *DNMT3A*, *TET2*, *ASXL1*, and *TP53* alterations were highly enriched in cases with multiple CH mutations (Figure 2D). Comparisons of the younger (<65 years) and older (≥65 years) populations revealed no clear differences in the age‐related C > T transitions^10^ (Figure 2E), but more frequent C > T transitions in the context of GCC in older patients (Figure 2F).

**FIGURE 2:**
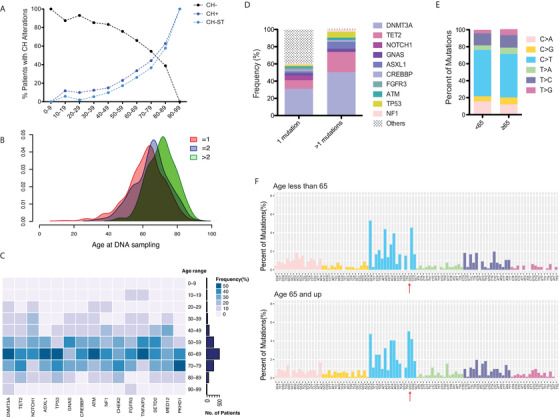
**Age‐associated accumulation of CH**. **A**, Age distributions of the entire cohort (N = 4544) with CH (CH+), with CH detected in solid tumor (CH‐ST), and without CH‐associated mutations in the blood (CH‐). Note a positive correlation with age in the CH+ or CH‐ST population, and a negative correlation with age in the CH‐ population. **B**, Accumulation of CH mutations with age. The median age at DNA sampling is 63, 66, and 72 for patients with one, two, and more than two CH mutations, respectively. **C**, Heatmap showing frequency distribution of patients within each age group across the top 15 recurrent CH genes. **D**, Patients displaying accumulation of CH alterations were enriched for *DNMT3A*, *TET2*, *ASXL1*, and *TP53* mutations. **E and F**, Mutational signatures of (**E**) single base substitutions and (**F**) substitutions with their nucleotide context are compared between different age groups. C > T transitions in the context of GCC are indicated by arrows

CH incidence was slightly higher in males than females (Table S1 and Figure S3A). Mutational analysis revealed increased *MED12* alterations and lower frequencies of *DNMT3A*, *NOTCH1*, *TP53*, *GNAS*, *ATM*, and *NF1* alterations in females (Figure S3B), but no apparent differences in mutational signatures between the sexes (Figure S3C and D). Mutational frequencies also varied by cancer types (Figure S4A). For instance, *DNMT3A* mutations were more common in lung and gastric cancers compared with colorectal cancer. Differences in mutational signatures (Figure S4B) might be attributable to the environmental forces underlying different cancer types, such as tobacco exposure, chemoradiotherapy, and viral infection.

CH‐associated mutations were detected in the respective tumors (CH‐ST) of 995 (76.5%) patients. Median VAF was 3.11% in the blood compared with 0.37% in the tissue (Figure 3A). High CH‐ST VAFs were associated with blood/leukocyte infiltration (Figure S5A). Note that 453 (25.9%) of CH‐ST mutations were functionally annotated using OncoKB as (likely) oncogenic. Importantly, we detected two *EGFR* kinase domain mutations (V742I in lung cancer and R776H in soft‐tissue sarcoma), as well as a *KRAS* mutation (F156L) and a loss‐of‐function mutation in *BRCA2* (Table S4). Tumor‐specific mutations in both CH+ and CH− samples showed significant overlap in VAFs with those of CH‐ST (Figure S5B).

**FIGURE 3:**
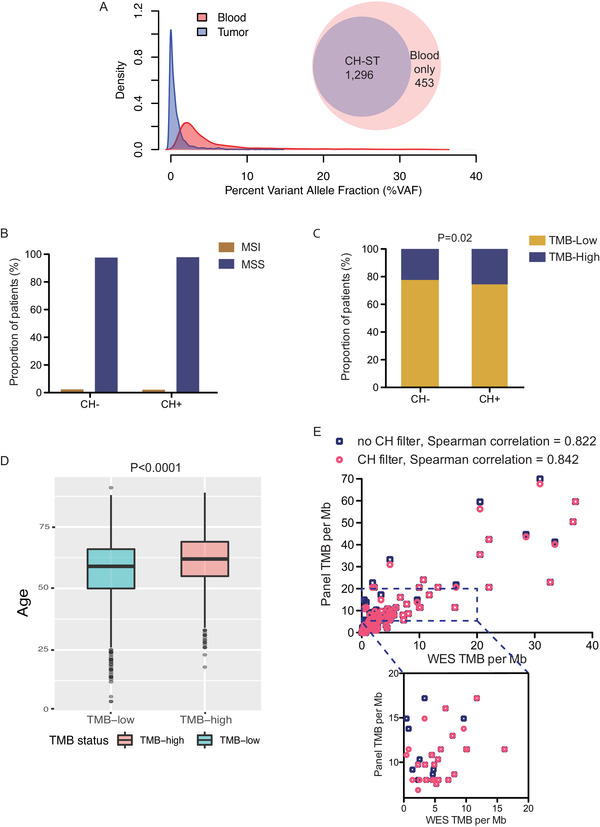
**Associations of immunotherapy biomarkers with CH. A**, Density of CH variant allele fraction in blood and tissue samples. Venn Diagram showing the fraction of CH variants detected in the respective solid tumor. **B**, The proportions of MSI patients between CH− and CH+ patients were not different (*P* = .65, chi‐square test). **C**, Higher proportion of TMB‐high patients in the CH+ compared with CH− populations (*P* = 0.02, chi‐square test). **D**, Association between high TMB and older age. **E**, Correlations of TMB between exome and panel sequencing with (circles) or without (squares) CH filtering in an independent cohort of 175 patients from whom tumor and matched normal samples were each sequenced

Finally, we evaluated the associations between CH and immunotherapy‐related markers. The proportion of microsatellite instability‐high patients was comparable between CH+ and CH− patients (2.1% vs 2.4%, *P* = .65, chi‐square test; Figure 3B). Using a cutoff of 10 mutations/Mb, a higher proportion of CH+ patients had high tumor mutational burden (TMB; 25.7% vs. 22.4%, *P* = .02, chi‐square test; Figure 3C), which can be explained at least in part by an aging‐associated increase in TMB (Student's *t* test, *P* < .0001; Figure 3D) and/or improper counting of CH variants. In an independent set of 175 patients with diverse cancers, we showed that incorporating CH filters improved the correlation between panel‐ and exome‐based TMB estimation (Spearman *ρ* = 0.842 with CH filter vs. 0.822 without; Figure 3E). TMB cut‐off levels can vary from 7.4 to 20 mut/Mb across different assays and cancer types. Of the 29 (16.6%) patients with TMB in the range of 7–20 mut/Mb, nine were CH‐positive (Figure 3E).

Our study provides the first report on CH and CH‐ST in a large population of Chinese cancer patients. Our findings, along with other recent work,^5^, ^6^, ^7^, ^8^ show high prevalence of CH and CH‐ST variants that challenges the interpretation of tumor‐only sequencing results. Given its highly diverse and nonrecurrent nature, it is essentially impractical to generate an extensive database encompassing all relevant CH variations. Consequently, sequencing of matched normal samples should be recommended in clinical practice, particularly for selection of targeted therapies. We also demonstrate for the first time that CH filtering is technically important for improving panel‐based TMB estimation. Further large‐scale prospective studies should evaluate the impact of CH filtering on immunotherapy outcome prediction.

## ETHICS APPROVAL AND CONSENT TO PARTICIPATE

Informed written consent was obtained from each subject or the subject's family member upon sample collection according to the protocols approved by the ethics committee of each hospital.

## CONFLICT OF INTEREST

Jiani C. Yin, Chenglong Na, Xue Wu, and Yang Shao are employees of Nanjing Geneseeq Technology Inc. All remaining authors have declared no conflict of interests.

## Supporting information

SUPPORTING INFORMATION

SUPPORTING INFORMATION

SUPPORTING INFORMATION

SUPPORTING INFORMATION

SUPPORTING INFORMATION

## Data Availability

The data that support the findings of this study are available from the corresponding authors upon reasonable request. The data are not publicly available due to privacy or ethical restrictions.
