# Supplementary material for: Comprehensive next‐generation profiling of clonal hematopoiesis in cancer patients using paired tumor‐blood sequencing for guiding personalized therapies
Source: Clin Transl Med. 2020 Nov 10;10(7):e222. doi: 10.1002/ctm2.222 (PMC7654627; doi:10.1002/ctm2.222)
Supplement: Supplementary file 1 — SUPPORTING INFORMATION [file CTM2-10-e222-s001.docx]

|  | All patients  N=4544 | CH+ patients  n=1301 | CH-ST patients  n=995 |
| --- | --- | --- | --- |
| Median age (range) | 60 (4-91) | 64 (13-91) | 65 (17-91) |
| <65 | 2832 (62.3%) | 623 (47.9%) | 456 (45.8%) |
| ≥65 | 1395 (30.7%) | 588 (45.2%) | 478 (48.0%) |
| Unknown | 317 (7.0%) | 90 (6.9%) | 61 (6.1%) |
| Sex |  |  |  |
| Male  Female  Unknown | 2522 (55.5%)  1825 (40.2%)  197 (4.3%) | 757 (58.2%)  483 (37.1%)  61 (4.7%) | 585 (58.8%)  366 (36.8%)  44 (4.4%) |
| Cancer Type |  |  |  |
| Lung cancer  CRC  Stomach cancer  Breast cancer  Hepatobiliary  Pancreatic cancer  Urogenital cancer  Skin cancer  Soft tissue sarcoma  Ovarian  Esophageal cancer  H&N  Uterine cancer  GIST  NEN  Cervical cancer  Thyroid cancer  Thymic cancer  Mediastinal cancer  lymphoma  CNS  Bone cancer  mesothelioma  Others  Unspecified | 1926 (42.4%)  1009 (22.2%)  404 (8.9%)  194 (4.3%)  136 (3.0%)  95 (2.1%)  94 (2.1%)  89 (2.0%)  72 (1.6%)  71 (1.6%)  62 (1.4%)  59 (1.3%)  32 (0.7%  31 (0.7%)  30 (0.7%)  24 (0.5%)  20 (0.4%)  9 (0.2%)  8 (0.2%)  5 (0.1%)  5 (0.1%)  4 (0.1%)  4 (0.1%)  24 (0.5%)  137 (0.3%) | 600 (46.1%)  286 (22.0%)  121 (9.3%)  41 (3.2%)  38 (2.9%)  28 (2.2%)  23 (1.8%)  20 (1.5%)  17 (1.3%)  18 (1.4%)  16 (1.2%)  14 (1.1%)  8 (0.6%)  9 (0.7%)  6 (0.5%)  8 (0.6%)  4 (0.3%)  1 (0.1%)  2 (0.2%)  1 (0.1%)  2 (0.2%)  2 (0.2%)  0  5 (0.4%)  31 (2.4%) | 472 (47.4%)  215 (21.6%)  90 (9.0%)  31 (3.1%)  29 (2.9%)  25 (2.5%)  16 (1.6%)  18 (1.8%)  12 (1.2%)  11 (1.1%)  12 (1.2%)  12 (1.2%)  4 (0.4%)  5 (0.5%)  2 (0.2%)  8 (0.8%)  1 (0.1%)  1 (0.1%)  1 (0.1%)  0  2 (0.2%)  1 (0.1%)  0  5 (0.5%)  22 (2.2%) |

Supplementary Table 1. Patient baseline characteristics

CH, clonal hematopoiesis; CH-ST, tumor-associated clonal hematopoiesis mutations; CRC, colorectal cancer; H&N, head and neck cancer; GIST, gastrointestinal stromal tumor; NEN, neuroendocrine neoplasms; CNS, central nervous system cancers.
