# Supplementary material for: Comprehensive next‐generation profiling of clonal hematopoiesis in cancer patients using paired tumor‐blood sequencing for guiding personalized therapies
Source: Clin Transl Med. 2020 Nov 10;10(7):e222. doi: 10.1002/ctm2.222 (PMC7654627; doi:10.1002/ctm2.222)
Supplement: Supplementary file 5 — SUPPORTING INFORMATION [file CTM2-10-e222-s005.pdf]

Supplementary Figure 1

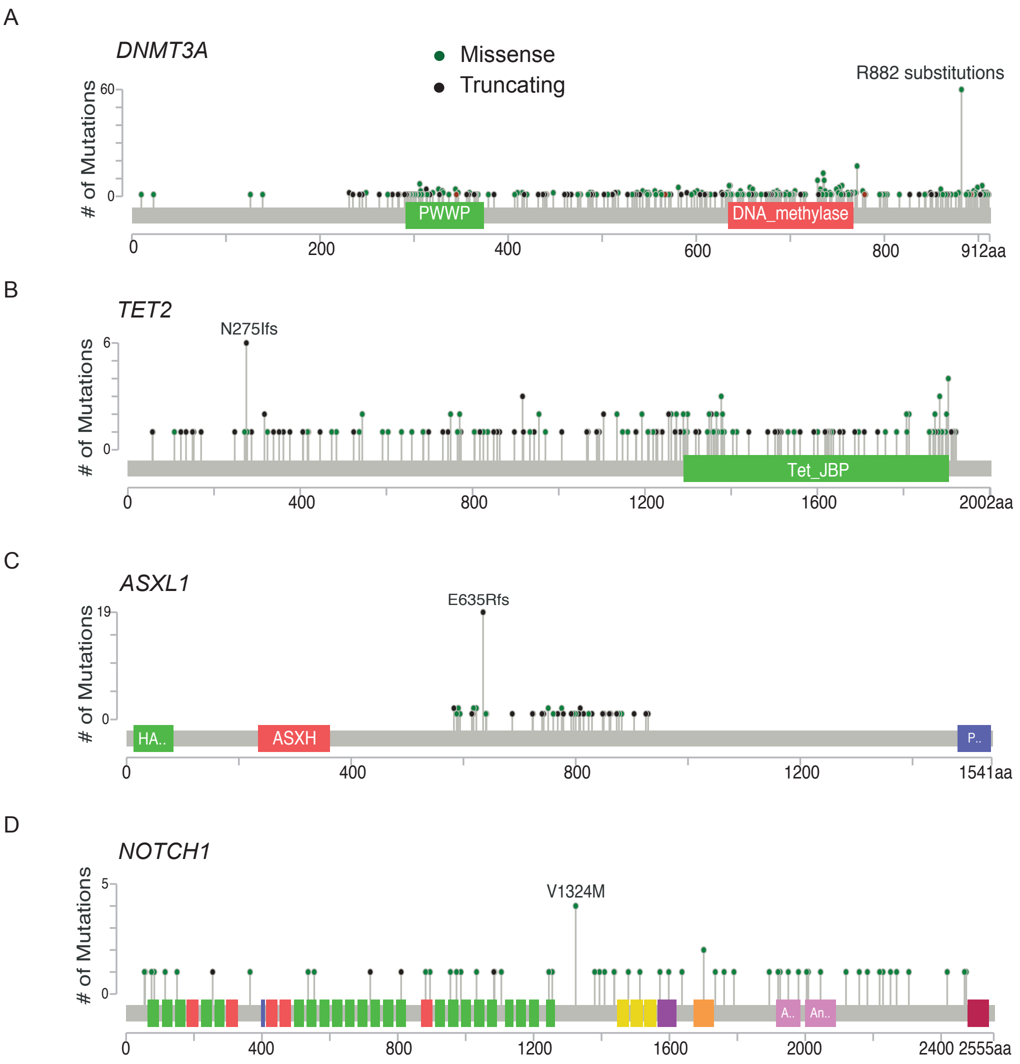

Supplementary Fig. 1. Graphical representations of mutations in top CH-associated genes. A-D, Distribution of CH-associated (A) *DNMT3A*, (B) *TET2*, (C) *ASXL1* and (D) *NOTCH1* alterations.

Supplementary Figure 2

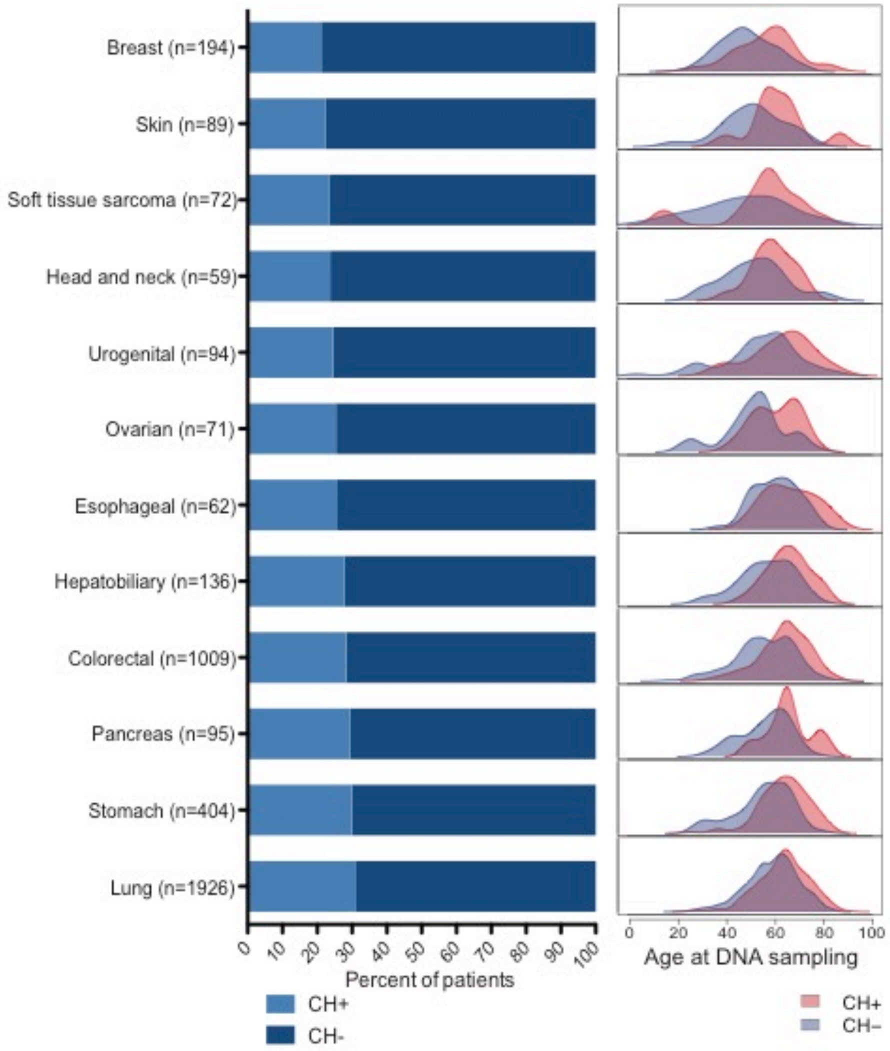

Supplementary Fig. 2. Percentage of patients with CH across the 12 most frequently assayed cancer types. Note the correlation with age at DNA sampling across different cancer types. Within each cancer, there is a rightward shift in the age of CH+ patients compared with CH-patients.

Supplementary Figure 3

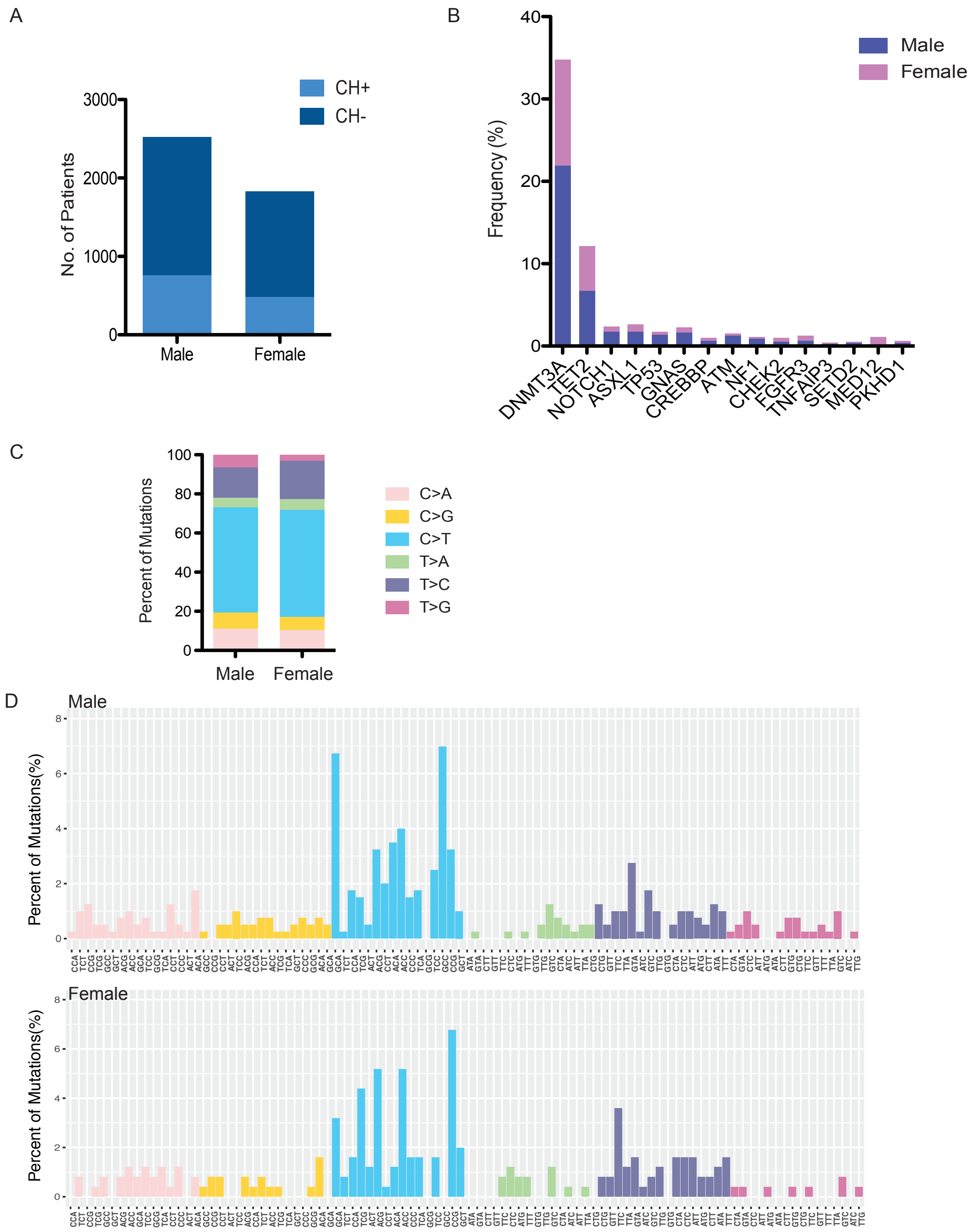

Supplementary Fig 3. No clear association between CH and sex. A, proportion of male and female patients with CH. B, Comparisons of proportion of male and female patients across the top 15 CH relevant genes. C-D, Mutational signatures of (C) single base substitutions and (D) substitutions with their nucleotide context.

Supplementary Figure 4

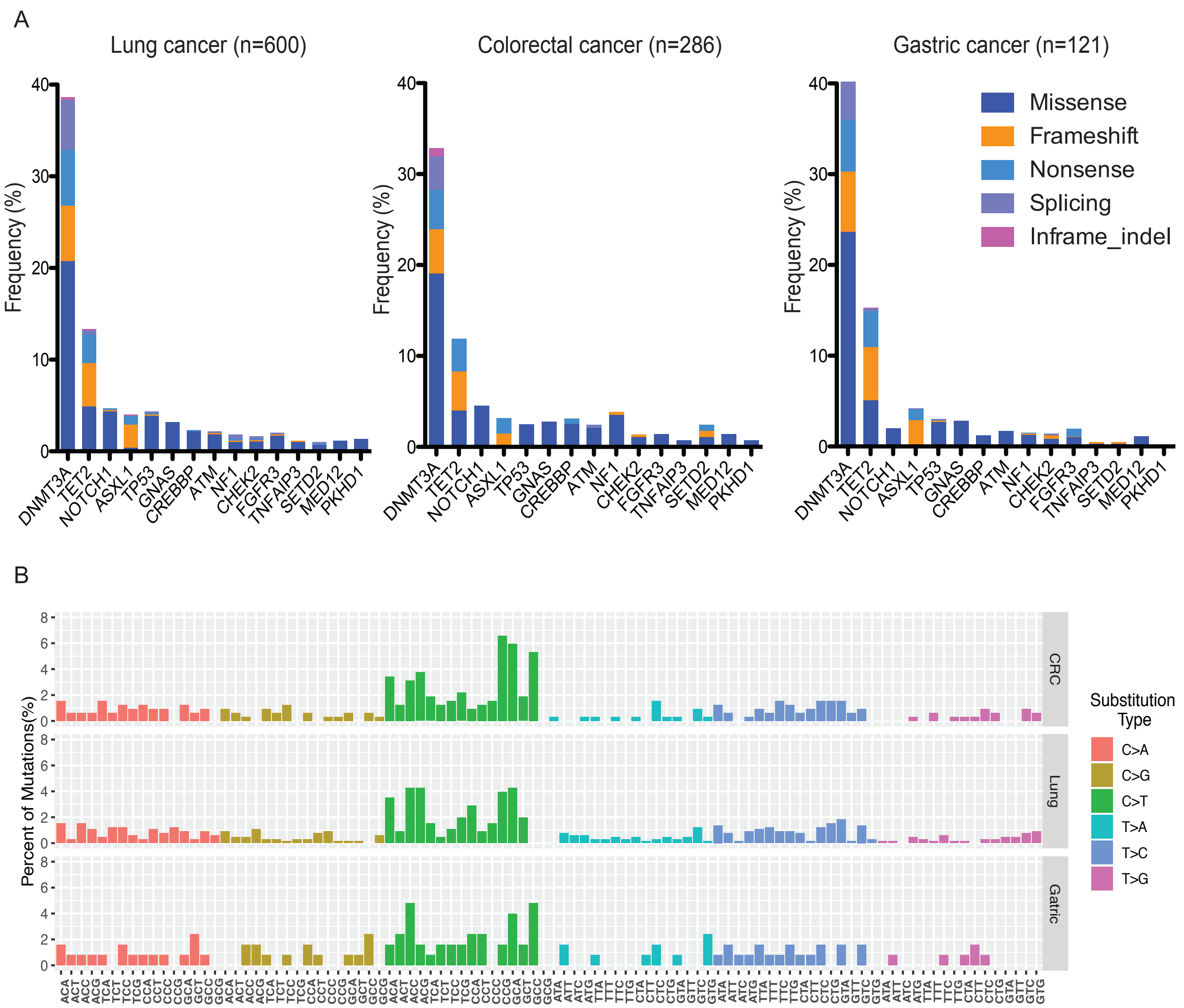

Supplementary Fig 4. CH-associated mutational landscape of patients with lung, colorectal and gastric cancers. A, Comparisons of distributions of mutations across the top 15 CH relevant genes in the three major cancer types. B, Mutational signatures of single base substitutions with their nucleotide context comparing the three major cancer types.

Supplementary Figure 5

A

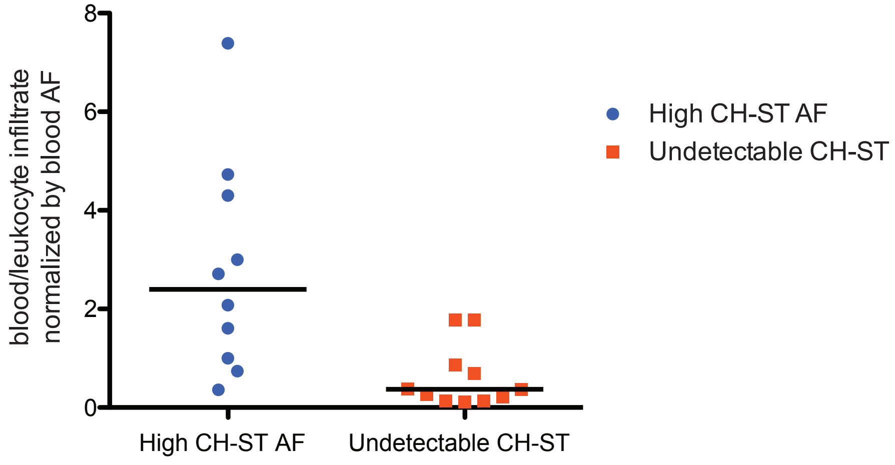

B

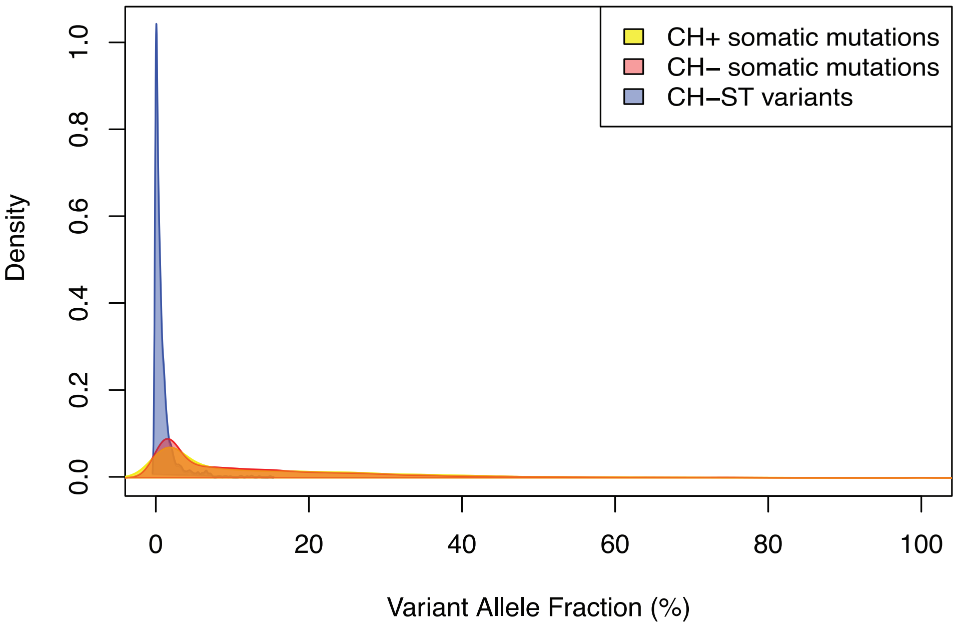

Supplementary Fig 5. Characteristics of CH-ST variants. A, Samples with high VAF of CH-ST and undetectable CH-ST were compared for their blood/leukocyte infiltration into the tumor tissue, following correction for VAFs of blood CH mutations. B, VAF distributions of somatic mutations and CH-ST variants.
